# Plasmer: an Accurate and Sensitive Bacterial Plasmid Prediction Tool Based on Machine Learning of Shared k-mers and Genomic Features

**DOI:** 10.1128/spectrum.04645-22

**Published:** 2023-05-16

**Authors:** Qianhui Zhu, Shenghan Gao, Binghan Xiao, Zilong He, Songnian Hu

**Affiliations:** a State Key Laboratory of Microbial Resources, Institute of Microbiology, Chinese Academy of Sciences, Beijing, China; b University of Chinese Academy of Sciences, Beijing, China; c School of Engineering Medicine, Beihang University, Beijing, China; d Beijing Advanced Innovation Center for Big Data-Based Precision Medicine, Interdisciplinary Innovation Institute of Medicine and Engineering, Beihang University, Beijing, China; e Sino-Danish College, University of Chinese Academy of Sciences, Beijing, China; University of Nebraska–Lincoln

**Keywords:** plasmid, chromosome, machine learning, shared k-mers, genomic features, bacteria, benchmark, k-mer, prediction tool, random forest

## Abstract

Identification of plasmids in bacterial genomes is critical for many factors, including horizontal gene transfer, antibiotic resistance genes, host-microbe interactions, cloning vectors, and industrial production. There are several *in silico* methods to predict plasmid sequences in assembled genomes. However, existing methods have evident shortcomings, such as unbalance in sensitivity and specificity, dependency on species-specific models, and performance reduction in sequences shorter than 10 kb, which has limited their scope of applicability. In this work, we proposed Plasmer, a novel plasmid predictor based on machine-learning of shared k-mers and genomic features. Unlike existing k-mer or genomic-feature based methods, Plasmer employs the random forest algorithm to make predictions using the percent of shared k-mers with plasmid and chromosome databases combined with other genomic features, including alignment E value and replicon distribution scores (RDS). Plasmer can predict on multiple species and has achieved an average the area under the curve (AUC) of 0.996 with accuracy of 98.4%. Compared to existing methods, tests of both sliding sequences and simulated and *de novo* assemblies have consistently shown that Plasmer has outperforming accuracy and stable performance across long and short contigs above 500 bp, demonstrating its applicability for fragmented assemblies. Plasmer also has excellent and balanced performance on both sensitivity and specificity (both >0.95 above 500 bp) with the highest F1-score, which has eliminated the bias on sensitivity or specificity that was common in existing methods. Plasmer also provides taxonomy classification to help identify the origin of plasmids.

**IMPORTANCE** In this study, we proposed a novel plasmid prediction tool named Plasmer. Technically, unlike existing k-mer or genomic features-based methods, Plasmer is the first tool to combine the advantages of the percent of shared k-mers and the alignment score of genomic features. This has given Plasmer (i) evident improvement in performance compared to other methods, with the best F1-score and accuracy on sliding sequences, simulated contigs, and *de novo* assemblies; (ii) applicability for contigs above 500 bp with highest accuracy, enabling plasmid prediction in fragmented short-read assemblies; (iii) excellent and balanced performance between sensitivity and specificity (both >0.95 above 500 bp) with the highest F1-score, which eliminated the bias on sensitivity or specificity that commonly existed in other methods; and (iv) no dependency of species-specific training models. We believe that Plasmer provides a more reliable alternative for plasmid prediction in bacterial genome assemblies.

## INTRODUCTION

Plasmids are autonomous extrachromosomal DNA molecules which are mostly circular and replicate independently with controlled copy numbers per cell. Plasmids usually carry nonessential genes that allow bacteria to adapt to specific environments and conditions (1). These genes, such as antibiotic or heavy metal resistance genes, are involved in alternative metabolic pathways or encode virulence factors (2). As plasmids can spread rapidly within and between bacterial populations (3, 4), the horizontal gene transfer mediated by plasmids is recognized as a major driving force for bacterial adaptation and diversification, which involves the mobilization of antibiotic resistance genes, the spread of degradative pathways, and the dissemination of virulence genes (5). Horizontal gene transfer mediated by plasmids has been proven to contribute to the forming of dangerous antibiotic-resistant bacterial strains, such as methicillin-resistant Staphylococcus aureus (MRSA) (6–8), which is a serious threat to human health. In addition, plasmids generally carry accessory genes, which are part of the accessory genome, the collection of genes that are present in one or more, but not all, genomes within a given clade (9). The accessory genes usually have diverse functions such as secondary metabolism and resistance, which are valuable in the development of industrial strains for bioremediation and biofertilization (10) and essential in gene cloning and exogenous protein expression. Therefore, the identification of plasmids in bacterial genomes is critical in a wide range of activities, such as understanding the evolution of bacteria through horizontal gene transfer between strains or species, monitoring the spread of antimicrobial resistance (AMR) genes in bacteria, unveiling the host-microbe interactions, and developing novel tools for industrial production.

Though there have been advances in short-read sequencing, the identification of plasmid contigs from fragmented contigs in genome assemblies remains a big challenge due to technical issues. Usually, successful identification of plasmids requires higher continuity of the assembled contigs. Contigs generated from short-read technologies were usually highly fragmented with large numbers of plasmid-like contigs, and thus it was hard to distinguish plasmids from chromosomes. The most recent PacBio or Nanopore long-read technologies could greatly improve the quality of assembled contigs and make it plasmid identification easy, but these technologies are still more expensive per sequencing depth than short-read sequencing and usually require very high starting DNA quality, which makes them inappropriate for regular studies, especially for large-scale pathogenic studies (11). Meanwhile, the identification of plasmids in those previous short-read genome assemblies from large amounts of existing short-read sequencing data is still critical when tracking the dynamics of plasmids. Moreover, plasmids are also involved in metagenomic studies, as there are good chances to understand the horizontal gene transfer mediated by plasmids in metagenomic samples, but these metagenomic assemblies are usually much more fragmented than those of single isolates, which creates greater challenges for properly identifying the plasmids. In addition, sequences from plasmids occasionally integrate into chromosomes, and sometimes plasmids also have essential chromosomal genes (12), which further increases the difficulty of correctly identifying plasmids from countless assembled contigs.

Up to now, many researchers have addressed the importance of plasmid identification in genome assemblies and have developed a variety of *in silico* methods. These methods can be mainly summarized into two categories: assembly-based methods and genomic features-based methods. The assembly-based methods attempt to identify plasmids using assembly information such as depth and assembly graphs from raw sequencing data. For example, plasmidSPAdes (13) assembles plasmid contigs based on depth variations between chromosomes and plasmids; PLACNET (14) is a graph-based plasmid reconstruction tool that creates a network of contig interactions from next-generation paired-end reads; Recycler and SCAPP predict plasmids based on the topology of the assembly graph (15, 16). Conversely, the genomic features-based methods attempt to identify plasmids from the assembled sequences by observing the content of sequences. For example, PlasmidFinder (17) uses BLAST to identify plasmids with similarity to known plasmids; Platon (18) predicts plasmids using replicon distribution score (RDS) together with other gene markers such as mobilization, conjugation, replication, *oriT*, and rRNA based on a decision tree-like workflow. Recently, machine learning methods based on k-mer frequencies, such as cBar (19), PlasFlow (20), mlplasmids (21), PlasClass (22), RFPlasmid (23), and Deeplasmid (24), have proven the feasibility of predicting plasmids using machine learning algorithms and shown good performance on novel plasmids. Meanwhile, PlaScope (25), PlasmidSeeker (26) and customized Kraken databases comprising known chromosomal and plasmid sequences of Klebsiella pneumoniae species complex (KpSC) (27) were developed to rapidly identify known plasmids in databases.

Nevertheless, every method has unique advantages and shortcomings. Due to complexities in assembly, methods based on the sequencing depth and topology of assembly graphs occasionally miss plasmids with similar copy numbers to chromosomes or falsely identify chromosomal contigs as plasmids which overlap at the ends or have a circular path in assembly graph (28). Methods based on BLAST-like searches can only identify known plasmids and usually make false predictions due to hard cutoffs of identity or coverage. Methods based on machine learning are capable of predicting novel plasmids, but most existing methods we tested have evident bias in sensitivity or specificity, which leads to either false-negative or false-positive predictions (18, 29). This means that in a conducted analysis, a choice must be made between conservative or aggressive classifications (29). Some methods, such as mlplasmids (21) and RFPlasmid (23), have different models for specific species or genera, which improved the overall accuracy. However, this is achieved at the cost of general versality, and such methods introduce new troubles in choosing appropriate models when unavailable. Notably, the performance of existing methods becomes unstable on short sequences of ≤10 kb (23), and many of them simply reject sequences of ≤1 kb (24). Considering that many contigs from short-read assemblies fall into the length below 1 kb, most existing methods may not be suitable for predicting plasmids in short-read genome assemblies.

To effectively predict plasmids in bacterial genome assemblies, especially for short-read-based assemblies, we proposed a novel method named Plasmer. The goal of Plasmer is to build a plasmid predictor with stable performance, balanced accuracy, sensitivity, and specificity that, most importantly, is capable of predicting on short-read based assemblies. To achieve this, Plasmer has learned from existing methods and has been designed to combine the advantages of k-mer databases, genomic markers, and machine learning algorithms. For each input sequence, Plasmer first queries the percent of shared k-mers with chromosome and plasmid databases and then extracts genomic markers, including replicon distribution score (RDS) (18), conjugation genes, mobilization genes, replication genes, AMR genes, *oriT* motifs, and rRNA genes. Finally, a random forest classifier is employed to predict the input sequence based on collected features. We have evaluated the performance of Plasmer on (i) sliding sequences of all complete bacterial genomes from NCBI GenBank with window sizes of 500 bp to 500 kb; (ii) assemblies of simulated sequencing reads from all complete genomes of Klebsiella pneumoniae, Enterococcus faecium, and Escherichia coli; and (iii) *de novo* assemblies of real sequencing data from previous mlplasmids (21), PlasFlow (20), Platon (18), and Deeplasmid (24) publications. Test results have shown that Plasmer has achieved both outperforming accuracy and solid stable performance on all tested input lengths above 500 bp, suggesting its applicability to highly fragmented assemblies. Notably, Plasmer has unbiased performance in both sensitivity and specificity, as well as the highest F1-score across all tested species, suggesting its applicability as a reliable and flexible plasmid prediction tool for bacterial genome assemblies. In addition, Plasmer also provides fast taxonomy classification of identified plasmids.

## RESULTS

### Selection of optimal k-mer size for k-mer databases.

To select optimal k-mer sizes for k-mer databases that separate plasmids and chromosomes as much as possible, we evaluated the distribution of k-mer-related features of training data using k from 17 to 27 (see Fig. S2 in the supplemental material). For plasmid-related databases (plasmid database p_ci1f1.0 and plasmid-minus-chromosome database pmr_ci1f1.0), any k-mer size can distinguish the plasmids and chromosomes; the percentage of intersecting k-mers decreased as the k increased and reached stability when k was 23 to 27. We then selected the middle number, k = 25, which relatively balanced the sensitivity and specificity, to build the plasmid database p_k25 and plasmid-minus-chromosome database pmr_k25. For chromosome-related databases (chromosome database r_ci1f0.1 and chromosome-minus-plasmid database rmp_ci1f0.1), the percentage of intersecting k-mers between the plasmid and chromosome input decreased when the k increased. However, the percentage of shared k-mers for chromosome input dispersed and spanned the full range when k was ≥22. Therefore, the target of selecting k-mer size for chromosome databases was to select a k size that distinguished plasmids and chromosomes while keeping the span width of chromosome input as narrow as possible. We found that when k was 17 to 19, the percentage of intersecting k-mers and the span width of chromosome input could be balanced. We then selected a k value of 18, the middle number from 17 to 19, which relatively balanced the sensitivity and specificity, to build chromosome database r_k18 and chromosome-minus-plasmid database rmp_k18. Finally, k values of 25 and 18, respectively, were selected for plasmid-related and chromosome-related databases for downstream model construction and plasmid prediction.

### Chromosomes and plasmids can be clearly distinguished by k-mer features and genomic markers.

In total, we sampled 1,573,876 sliding sequences, including 900,000 chromosomal sequences and 673,876 plasmid sequences for model training. To demonstrate that the percentage of shared k-mers can distinguish chromosomal and plasmid sequences, we performed a principal-coordinate analysis (PCoA) using the k-mer-related features, including r_k18, p_k25, rmp_k18, and pmr_k25. The result (Fig. S3) illustrated that the chromosome (lake-green) and plasmid (orange) sequences can be clearly distinguished based on the k-mer features, with PCoA1 (horizontal) accounting for 89.96% of variance and PCoA2 (vertical) accounting for 5.84% of variance.

To further demonstrate that features from genomic markers can also distinguish chromosomes and plasmids, the distribution of all features in the training set were plotted. In addition to k-mer-related features, the features that showed the second-most-evident differences were RDS-derived features such as RDSMaxScore (maximum RDS score of genes), RDSAvgScore (average RDS score of genes on input sequence), and rdsBias (the RDS bias of each sequence) (Fig. S4). Other gene- or motif-related features showed supplementary contributions to the separation of chromosomes and plasmids.

### The random forest model had excellent performance in classifying plasmid and chromosomal sequences.

**FIG 1:**
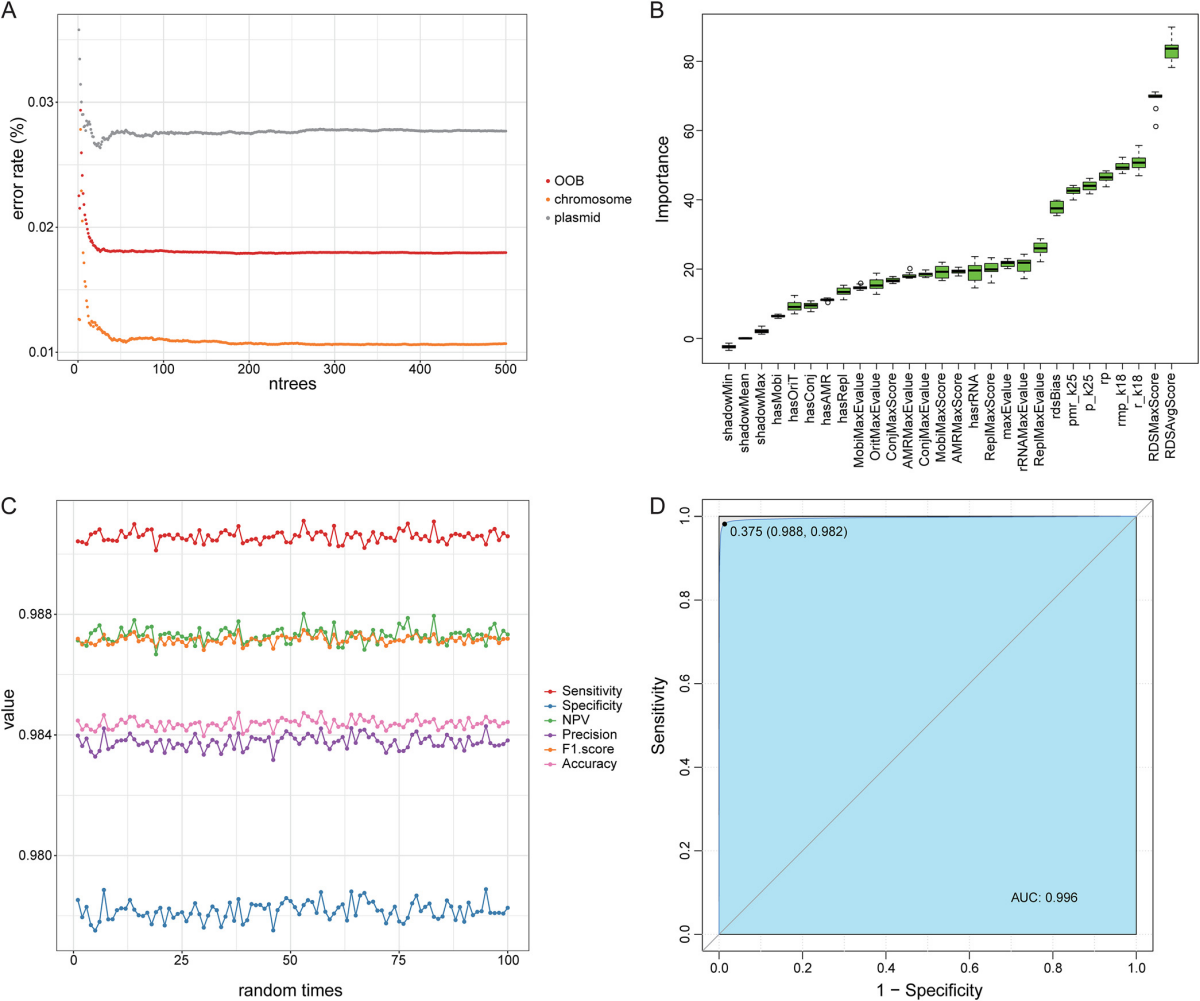
The performance of the classified model based on random forest analysis. (A) The estimation of the number of trees in the model. The *x* axis represents the number of trees in the model; the *y* axis represents the error rate of the model. OOB, out of bag. (B) The importance of all features. The *x* axis represents all the features; the *y* axis represents the importance. Green means the feature is important for classifying. (C) The performance indicators of the model during 100 random time points. Different colors represent different indicators. (D) The ROC curve of the model. The *x* axis represents 1 specificity; the *y* axis represents the sensitivity. The area under the curve (AUC) is 0.996.

Before model training, we evaluated the number of trees and the importance of all features in the random forest. The error of the random forest model gradually converged when the number of trees grew above 200 (Fig. 1A), confirming the feasibility of our selection of the number of trees (*n*trees = 500). Meanwhile, the top important features were RDS-related features, followed by four k-mer related features, while gene- and motif-related features fall behind, confirming the pattern observed in distribution plots. Since all features were important for the model (Fig. 1B), all features were used for training. After 100 repetitions of random sampling and random forest modeling, the accuracy was around 98.5%, and the sensitivity reached around 0.99; all other metrics, including F1-score, precision, and negative predictive value (NPV) were above 0.98, and specificity approached 0.98. (Fig. 1C). Furthermore, the AUC of our model has reached to 0.996 (Fig. 1D). In total, the above-described results indicate that the model had excellent performance.

### Performance comparisons of different input lengths using remaining sliding sequences of all complete bacterial genomes in NCBI.

**FIG 2:**
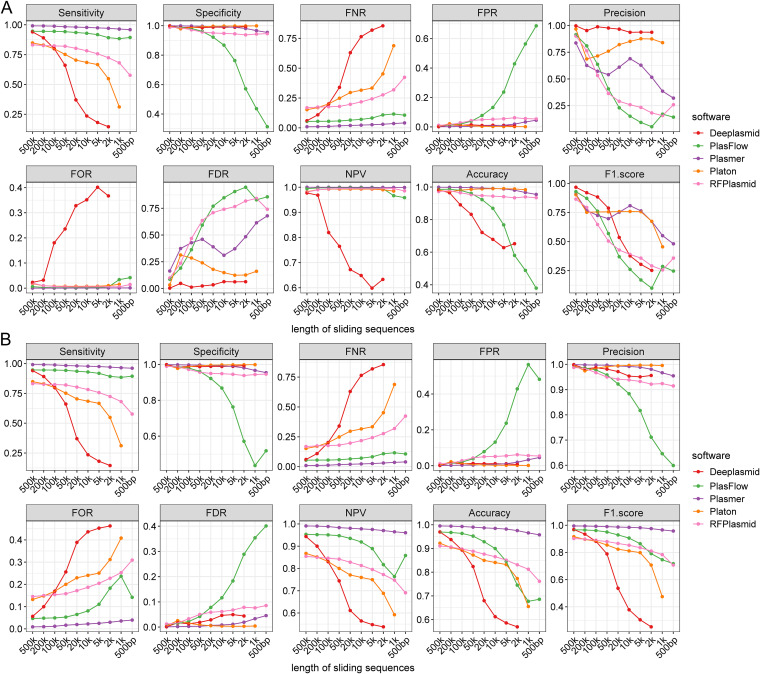
The performance of Plasmer and other methods for the remaining sliding sequences with different lengths of 31,897 complete bacterial genomes in NCBI. Each subpanel represents one indicator, such as sensitivity. The *x* axis represents lengths of the sequences; the *y* axis represents the values of indicators. Different colors represent different indicators. (A) The performance on all remaining sliding sequences. (B) The performance on resampled sliding sequences with balanced numbers of chromosomes and plasmids.

To evaluate the performance and applicability of Plasmer on different input lengths, we tested 31,897 complete bacterial genomes, excluding the sequences included in training sets with different sliding window sizes (500 kb, 200 kb, 100 kb, 50 kb, 20 kb, 10 kb, 5 kb, 2 kb, 1 kb, and 500 bp). Due to the lack of a general model, mlplasmids was not included in this comparison. It is worth noting that Plasmer achieved the highest sensitivity, NPV, and F1-score; specificity, accuracy, and false-positive rate (FPR) comparable to those of Platon (18); and the lowest false-negative rate (FNR) and false-omission rate (FOR) in all tested lengths (Fig. 2A and Table S8). Notably, Plasmer had constantly stable performance on sensitivity, specificity, and accuracy, which stayed above 0.95 even if the input length decreased to 500 bp. In comparison, Platon had excellent accuracy and specificity and the lowest FPR, but the sensitivity and FNR evidently dropped as the length decreased. Similar to Platon, the generic model RFPlasmid (23) had good specificity and accuracy but, still, descending sensitivity. PlasFlow (20) had acceptable performance on longer sequences, but the accuracy and specificity decreased below 0.8 when sequences were shorter than 10 kb. Deeplasmid (24) seemed to have better performance identifying chromosomes than plasmids. Overall, most of the methods achieved excellent overall specificity and accuracy, but the performance on other metrics such as sensitivity was very different as the input length decreased, as the sensitivity was identical to the “plasmid accuracy,” which means the drop on the sensitivity could miss plasmids (false negative) in prediction. Compared to other methods, Plasmer is less affected by the input length. and its sensitivity stayed above 0.95, which means it is less prone to false-negative predictions. In addition, some methods had limits on input length. For example, Deeplasmid failed to predict on lengths of ≤1 kb, Platon excluded all input of <1 kb. Only Plasmer, RFPlasmid, and PlasFlow could make predictions when the length dropped to 500 bp.

Since sliding windows are small fragments of assembled contigs with identical lengths, it creates a huge number of sequences when the window size gets small. In our results, the number of sliding sequences increased dramatically as the length decreased; we therefore must pay attention to the unbalanced number of sequences between chromosomes and plasmids. According to the prevalence (Table S8), there could be over 100 times more chromosome sequences than plasmids, which was far more than that in real genome assemblies. In such situations, the number of false-positive predictions was exaggerated and metrics such as precision, accuracy, false-discovery rate (FDR), and F1-score were not credible for the model’s performance but were biased toward the sample size. Therefore, we carried out a more convincing balanced test by down-sampling the chromosome sequences to the number of plasmids for each length. On the balanced test, Plasmer outperformed most of other methods on most of the metrics, including the highest sensitivity, accuracy, NPV, and F1-score and the lowest FNR and FOR (Fig. 2B and Table S9). Even though Platon and Deeplasmid had comparable performance on specificity, precision, FPR, and FDR, their sensitivity and accuracy dropped quickly as the input length decreased. Plasmer showed constantly stable performance on all tested lengths, while the performances of other methods greatly dropped on short lengths.

In summary, both tests on sliding sequences from all complete bacterial genomes demonstrated that Plasmer had more stable performance on a wide range of lengths than the other tools and was the first one applicable to 500-bp lengths, which made Plasmer a stable, accurate, and sensitive tool for plasmid prediction.

### Performance comparison of simulated reads of K. pneumoniae, E. coli, and E. faecium.

**FIG 3:**
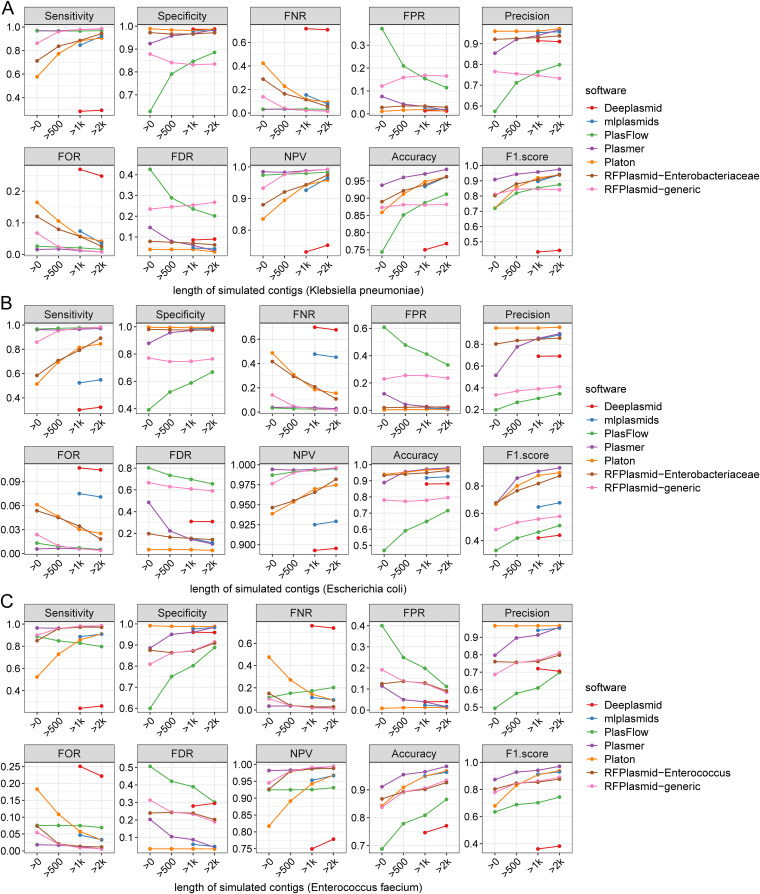
The performance of Plasmer and other methods for contigs assembled by simulated reads of Klebsiella pneumoniae, Escherichia coli, and Enterococcus faecium. Each subpanel represents one indicator, such as sensitivity. The *x* axis represents the lengths of the sequences; the *y* axis represents the values of indicators. Different colors represent different indicators. (A) The 552 complete genomes of Klebsiella pneumoniae. (B) The 1,718 complete genomes of Escherichia coli. (C) The 123 complete genomes of Enterococcus faecium.

To evaluate Plasmer on assembled contigs, we simulated and reassembled sequencing reads of 552 complete genomes of K. pneumoniae, 1,718 complete genomes of E. coli, and 123 complete genomes of E. faecium (Fig. S1D). For K. pneumoniae, we labeled 22,746 plasmid and 49,433 chromosome contigs. For E. coli, we labeled 49,036 plasmid and 367,039 chromosome contigs. For E. faecium, we labeled 9,490 plasmid and 20,357 chromosome contigs. To also evaluate the impact of length, we made gradient statistics by filtering short contigs to different lengths (unfiltered, 500 bp, 1 kb, and 2 kb). According to Table S10, we found that the prevalence of K. pneumoniae and E. faecium was around 0.3, and that of E. coli was only ~0.15, which led to the disabled precision and FDR; the accuracy and F1-score were also impacted. In comparison, Plasmer continued to have the highest accuracy and F1-score and balanced sensitivity and specificity above 500 bp for all three species (Fig. 3 and Table S10). Although the specificity and precision of Platon were higher than those of Plasmer, its sensitivity dropped below 0.6 and remained below 0.8 after filtering to 500 bp, which created the risk of missing many plasmid sequences. The generic model of RFPlasmid seemed to perform with higher sensitivity than specificity, which created the risk of falsely predicting chromosomes as plasmids. The specific model of RFPlasmid performed with higher specificity, F1score, accuracy, and precision and lower sensitivity than the generic model. The mlplasmids model was designed for sequences longer than 1 kb, and for the *E.coli* sequences, the sensitivity was lower than 0.6 and the F1-score was lower than 0.7; for the other species, the performance was comparable with that of Platon. Due to the unbalance of sensitivity and specificity, the F1-scores of Deeplasmid were lower than those of the other software, and it was only available for sequences longer than 1 kb. In summary, Plasmer is applicable for contigs assembled by simulated sequencing reads with the most balanced performance. When ignoring contigs shorter than 500 bp, Plasmer has sensitivity, specificity, and accuracy higher than 0.95 and FPR and FDR lower than 0.1.

### Performance comparisons using real sequencing data.

**FIG 4:**
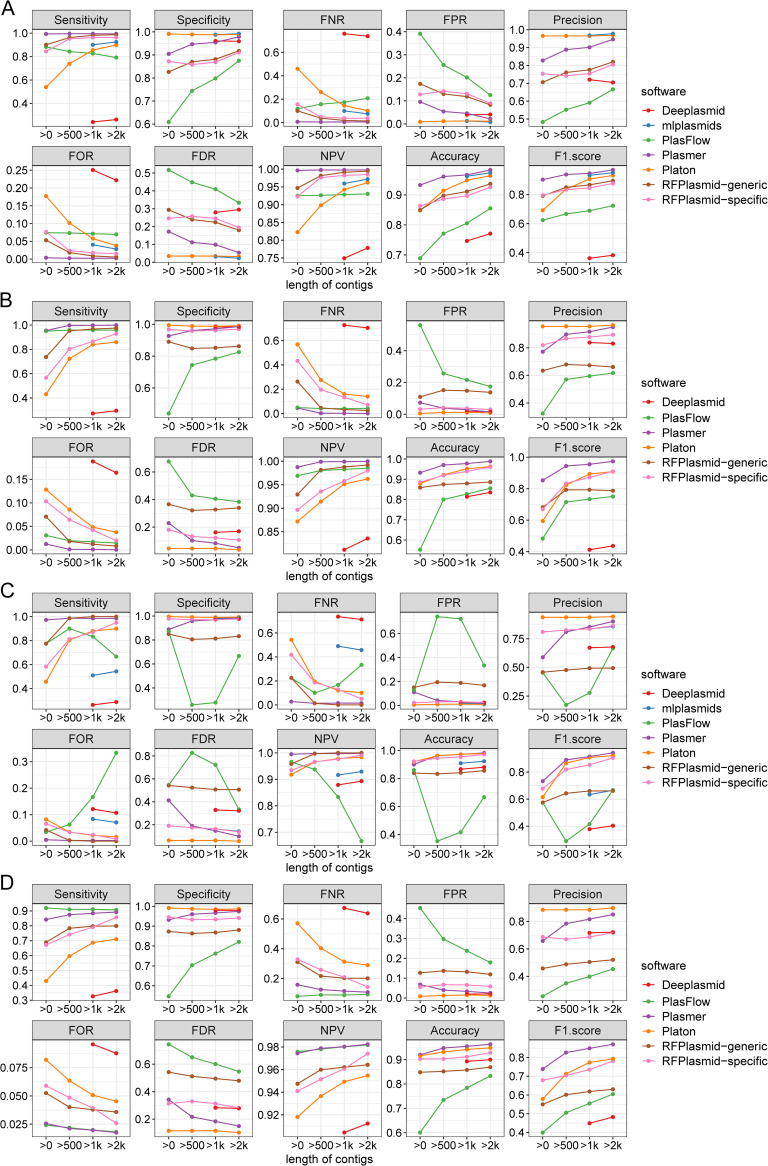
The performance of Plasmer and other methods for contigs assembled by real sequencing reads. Each subpanel represents one indicator, such as sensitivity. The *x* axis represents the lengths of the sequences; the *y* axis represents the values of the indicators. Different colors represent different indicators. (A) The sequencing reads of 41 isolates used in the mlplasmids (21) publication. (B) The sequencing reads of 40 isolates used in the PlasFlow (20) publication. (C) The sequencing reads of 21 new isolates used in the Platon (18) publication. (D) The performance of Plasmer and other methods on the assembled contigs of 535 recently published genomes of multiple species.

**(i) Performance on isolates with reference genomes.** To evaluate the performance of Plasmer on real sequencing data, we assembled the raw sequencing data of 41 E. faecium isolates used in mlplasmids publications and 40 isolates from various species used in PlasFlow publications (Tables S5 and S6). The reference genomes available in NCBI GenBank were used for validation. Because the prevalence was lower than 0.3 (Table S11), which means there were far more chromosomal sequences than plasmid sequences in the actual short-read assemblies, the precision, FDR, accuracy, and F1-score were biased. Despite this, Plasmer continued to have the highest sensitivity, accuracy, F1score, and NPV and lowest FNR and FOR (Fig. 4A and B; Table S11), which was similar to benchmarks of simulated reads. Platon had excellent specificity, but the unideal sensitivity dragged down its overall performance. Conversely, RFPlasmid had excellent sensitivity, but the specificity was not ideal. It was interesting that mlplasmids had comparable performance with Plasmer for the 41 samples from the mlplasmids publication, but the sensitivity was lower than 0.6 for the 21 samples in the Platon publication. Due to distinct species in 40 samples in the PlasFlow publication, mlplasmids was not used in this comparison. Deeplasmid and PlasFlow underperformed compared to their normal capability on such tests. In summary, these tests demonstrated that Plasmer is capable of recovering plasmid or chromosome fragments from short-read genome assemblies with better overall performance than that of available methods for real sequencing data.

**(ii) Performance for *de novo*-assembled sequences.** To evaluate the performance of Plasmer on *de novo*-assembled sequences which were not included in the training data, we downloaded raw sequencing data of 21 isolates used in the Platon manuscript with both long reads and Illumina short reads available for each isolate (Table S7). We assembled the Nanopore long reads and labeled the chromosome and plasmids (Table S12 and S13) and then used them as the reference genomes to validate the performance on the corresponding Illumina-only assemblies. Meanwhile, as these assemblies were Escherichia coli species, which was common in the training data, we also tested the Yersinia ruckeri isolate used in the Deeplasmid manuscript (Table S7), which was absent in the training data. Furthermore, to validate the performance on new genomes of multiple species, we also tested 535 complete genomes recently published in NCBI (between 1 July 2022 and 15 April 2023) (Table S14). The Illumina-only assembled contigs were labeled as chromosome or plasmid and predicted using Plasmer and other tools.

As shown in Fig. 4C and Table S11, Plasmer still achieved the highest F1-score, although the prevalence was only ~0.15. After sequences shorter than 500 bp were removed, the specificity and accuracy of Plasmer reached above 0.95. Plasmer had the highest F1-score, sensitivity, and NPV and lowest FNR and FOR on unfiltered contigs. Platon had the highest specificity and comparable accuracy with Plasmer, with an F1-score second to that of Plasmer. However, Platon had almost the second-lowest sensitivity, which indicated that many plasmid sequences were identified as chromosomes. Despite the generic model of RFPlasmid having sensitivity, NPV, FNR, and FOR comparable to those of Plasmer, the much lower specificity and higher FPR resulted in the much lower accuracy and F1-score than those of Plasmer and Platon. The *Enterobacteriaceae* model of RFPlasmid had higher specificity, F1-score, and accuracy and lower sensitivity and FDR than the generic model. Although the specificity of Deeplasmid was comparable with that of Plasmer for sequences longer than 1 kb, its performance was undermined by the lowest sensitivity and F1-score. Mlplasmids had excellent specificity but unideal sensitivity on contigs longer than 1 kb. Due to that the sequences predicted as unclassified were not included in the calculation of metrics, the performance of PlasFlow fluctuated greatly and was not optimal.

Meanwhile, benchmarks on the Yersinia ruckeri isolate (Table S11) and 535 recently published genomes of multiple species (Fig. 4D and Table S15) showed that Plasmer had a slight performance drop with new sequences, but it still had balanced sensitivity and specificity with the best F1-score and accuracy. This demonstrated that Plasmer could identify plasmids in actual assembled sequences, even if they were from uncommon species or species new to Plasmer. We also saw that RFPlasmid performed good if specific models were used. However, this could also limit its applicability for sequences from uncommon species.

According to the results, Plasmer has proven its applicability and outstanding overall performance in predicting plasmid sequences from *de novo*-assembled contigs of both common and uncommon species.

## DISCUSSION

To effectively identify plasmid contigs in bacterial genome assemblies, we proposed Plasmer, an accurate and sensitive bacterial plasmid prediction tool. Plasmer is essentially a two-class classifier separating plasmid and chromosome contigs in bacterial genome assemblies, based on a random forest machine learning model by combining the advantages of shared k-mers and genomic features. We benchmarked Plasmer together with other mainstream methods using different data types, including sliding sequences of complete genomes for performance on different lengths, and assemblies from both simulated reads and real sequencing data for applicability in real situations. The results have shown that Plasmer has outperforming accuracy, balanced sensitivity and specificity, and the highest F1-score for multiple species, making Plasmer an optimal alternative for predicting both chromosome and plasmid sequences. Meanwhile, Plasmer also showed its applicability to short sequences below 1 kb, which not only demonstrated that Plasmer was suitable for single-isolate genome assemblies, but also suggested its potential in predicting plasmids in highly fragmented bacterial assemblies, such as metagenomic assemblies. However, the performance on short sequences below 500 bp was not stable, as they rarely contain genes and sometime include insertion sequences (ISs) or repetitive sequences. Therefore, we recommend 500 bp as the minimum length for the input sequences for reliable predictions, but users could tune it down as needed.

Unlike other k-mer-based machine learning methods, Plasmer did not directly observe and train on the k-mer frequencies. It used the percentage of shared k-mers with chromosome and plasmid databases to measure the relative bias of sequences. Most of the other k-mer-based machine learning methods extracted k-mers with a relatively small k length, usually 5 to 7. This was due to exponential growth of number of k-mer indexes when a larger k length was used, which greatly increased the computational complexity for any learning model. However, small k size is obviously not unique enough to distinguish chromosomes and plasmids. In our test (Fig. S2), even with a k value of 17, the intersections between chromosomes and plasmids were still high. Therefore, models with small k-mers, such as mlplasmids and RFPlasmid, could perform better within a genus or species. In contrast, the percentage of shared k-mers proposed in Plasmer allowed the use of larger k values (currently k of 18 for chromosomes and k of 25 for plasmids) for higher sensitivity with only few features. Fewer features also allowed a larger training set for Plasmer and therefore more accurate predictions. In practice, we fed over 1 million sequences from all complete bacterial genomes in NCBI GenBank as the training set, which further enabled Plasmer, a generic classifier without training for specific species. Meanwhile, Plasmer was trained on mixed lengths including 500 kb, 200 kb, 100 kb, 50 kb, 20 kb, 10 kb, 5 kb, 2 kb, and 1 kb, which enabled Plasmer to give stable performances on a wide range of lengths. In contrast, PlasFlow was trained using only 10-kb sequences; its performance was greatly affected by input length.

In addition to k-mer features, Plasmer also integrated genomic markers, such as RDS, conjugation, mobilization, replication, *oriT*, and rRNA for a higher accuracy. The feasibility of these genomic markers has been verified by many existing methods, such as Platon, RFPlasmid, and Deeplasmid. In these methods, the presence of genomic markers was determined by searching input sequences with a series of databases and setting hard cutoffs. However, things were very different in Plasmer. For example, in Platon, the decisions were made by hard cutoffs on RDS depending on the applied filter mode (sensitivity, accuracy, and specificity), where the default is ‘accuracy mode’ with an RDS of ≥0.4 for plasmids and RDS of ≤−7.7 for chromosomes. In RFPlasmid, the presence of replication genes was determined by the BLASTP E-value of ≤1e-30. In contrast, Plasmer used the original scores and E-value as features in training, which enabled the quantitative description of genomic markers and let the model make proper decisions. As demonstrated (Fig. 1B), the quantitative genomic features had evidently higher importance than the binary features from hard cutoffs (such as RDSAvgScore versus rdsBias), which certainly contributed more to the overall performance.

Meanwhile, the core algorithm was also a decision role among tested methods. The random forest classifier used by RFPlasmid and Plasmer is well known to be sensitive, less prone to overfitting, and suitable for supervised classification (30, 31). In Platon, decisions were made based on conditions one after another, which was substantially a “decision tree.” In fact, decision trees are basic components in random forest methods. They work in similar mechanisms, but in random forests, the final decision is made by voting of all contained trees. The main shortcoming of Platon was the single decision tree with hard cutoffs, which easily neglected plasmids without sufficient evidence above hard cutoffs. Coincidently, chromosome contigs rarely contain plasmid-related marker genes. Highly fragmented plasmid contigs without complete genes could be easily identified as chromosomes, but chromosome fragments were not that easily identified as plasmids, which is asymmetrical. This could be the explanation for the relatively low sensitivity and high specificity of Platon. Unlike decision trees and random forests, Deeplasmid and PlasFlow are based on a neural network, while mlplasmids is based on a support-vector machine (SVM). These algorithms were also excellent classifiers, but the neural networks generated probabilities rather than making direct decisions; therefore, a trade-off on probability is necessary to make a prediction. For example, the cutoff on probability was 0.7 for PlasFlow and 0.5 for Deeplasmid by default, but users still have to find a good value that performs best on their own data.

In addition to the selection of algorithms, the selection and utilization of features also greatly contributed to the final performance. For example, Plasmer and RFPlasmid shared the same random forest core algorithm, but Plasmer still achieved better overall performance, which benefited from the selection of k-mer-based features and several improvements on the use of genomic marker-based features.

Nevertheless, Plasmer has its limitations. First, Plasmer is a supervised random forest classifier which has the common limitations of supervised machine learning approaches. Despite current tests showing that Plasmer performs well on new sequences (Table S11), Plasmer still can make an incorrect prediction if one sequence has very distinct signatures, such as newly identified species and mega-plasmids. To resolve this, the databases, training model, and algorithm of Plasmer require routine updates. Second, Plasmer is a two-class classifier; however, many sequences can horizontally transfer between chromosome and plasmid, such as AMR genes. These transferable sequences were substantially identical in both chromosomes and plasmids, creating an “ambiguous” class. Currently, as a supervised classifier, Plasmer was unable to identify them due to the difficulty of assigning the proper “ambiguous” label to sequences when training. Nevertheless, we are planning to build a multiclass classifier by self-training or generative approaches that recognize the “ambiguous” for horizontal gene transfer (HGT) studies. Third, limited by the training data, Plasmer currently only supports bacterial sequences, despite Plasmer showing better performance on short contigs, which indicates its potential to identify plasmids in assemblies of complex metagenomes. However, real metagenome assemblies are usually mixtures of vectors and hosts, such as bacteria (including *Mycoplasma* and *Chlamydia*), archaea, fungi, viruses, protozoa, and humans. It could be possible to classify the sequences in a metagenome assembly by constructing the k-mer and gene databases for fungi, viruses, and other microbes and designing a more complicated machine-learning model for multiple classes, which was also our long-term goal.

In summary, Plasmer is a novel approach for bacterial plasmid prediction which has outperforming accuracy and balanced sensitivity and specificity, with the highest F1-score and stable performance even on short sequences above 500 bp. We believe that the advantages of Plasmer will make it a more reliable alternative than mainstream methods and boost related studies such as horizontal gene transfer and microbial resistance. In the future, we plan to develop Plasmer into a universal plasmid and HGT classifier which is applicable for multiple microbes in metagenome assemblies.

## MATERIALS AND METHODS

### Overview of the Plasmer workflow.

**FIG 5:**
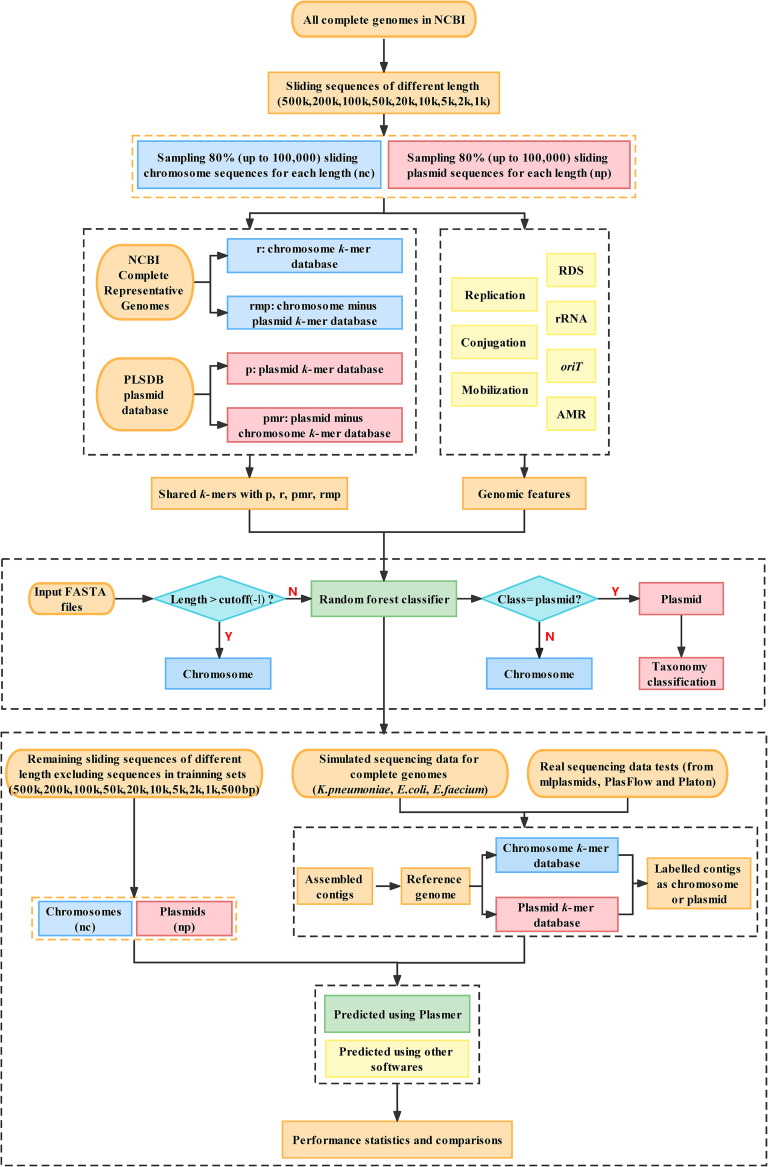
The workflow of Plasmer. The nc and np boxes represent the chromosomes and plasmids, respectively, of 31,897 complete bacterial genomes in NCBI. The r, p, pmr, and rmp boxes represent the k-mer database from chromosomes of representative genomes, the k-mer database from plasmids of PLSDB, the plasmid-unique k-mer database (plasmid database minus k-mers from chromosomes of representative genomes), and the chromosome-unique k-mer database (representative chromosome database minus k-mers from PLSDB), respectively.

The workflow of Plasmer (Fig. 5) consisted of three stages: (i) data preparation and construction of k-mer databases, (ii) extraction of sequence features and construction of the machine learning model, and (iii) prediction of input sequences using the machine learning model. First, Plasmer checked the length of each input sequence. Sequences longer than a given cutoff (500 kb by default) were directly labeled as chromosome sequences. This was because long sequences (>500 kb) rarely originate from plasmids, as those often encode genetic features hindering the assembly of larger sequences, for example, transposons and integrons (18). Skipping such long sequences could save a lot of run time. Second, the percentage of shared k-mers with databases of known plasmids and chromosomes for each input sequence was calculated. Third, higher-level genomic markers such as RDS, conjugation genes, *oriT* motifs, and rRNA genes were integrated by Plasmer to increase overall performance. Next, a random forest classifier was trained using about 1 million sequences sampled from NCBI complete bacterial genomes, and the performances were benchmarked on the remaining sequences from NCBI complete bacterial genomes. Finally, the origin of the sequences was predicted using the constructed classifier, and the performance was evaluated on genome assemblies from both simulated and real sequencing data.

### Data preparation and construction of k-mer databases.

To construct of the chromosome k-mer databases, we downloaded a total of 2,935 complete bacterial genomes marked as “representative” in NCBI GenBank and excluded all sequences with the keyword “plasmid” (Table S1). Conversely, to construct the plasmid k-mer databases, we downloaded and used 34,513 plasmid sequences from PLSDB (32). KMC (v3.2.1) (33) was used to count the k-mers of chromosome and plasmids sequences with the parameter -fm -ci1, and Kmer-db (v1.9.2) (34) was used to build k-mer database from KMC output. Chromosome databases were built with the parameter f = 0.1 for a min-hashed database to reduce the size of the k-mer database, while the plasmid databases were built with default parameters. Next, we also built the plasmid-unique k-mer database PMR (plasmids database minus k-mers from chromosomes of representative genomes) and the chromosome-unique k-mer database RMP (representative chromosome database minus k-mers from PLSDB [32]) using the kmers_subtract function of kmc_tools (v3.2.1) (33) in conjunction with Kmer-db (v1.9.2) (34) (Fig. S1A). To determine the optimal k-mer size of chromosome and plasmid databases, we tested with continuous k from 17 to 27 and plotted the distribution of the percentage of shared k-mers for training data (see Results and Fig. S2). According to the result, we selected a value of 18 for chromosomes and a k value of 25 for plasmids, which accordingly selected the r_k18, p_k25, rmp_k18, and pmr_k25 databases, representing chromosome k-mer database, plasmid k-mer database, chromosome-unique k-mer database, and plasmid-unique k-mer database, respectively, for further analysis.

### Extraction of k-mer features and higher-level genomic markers of sequences.

For each input sequence, two catalogues of features were extracted: one is the percent of shared k-mers with plasmid- and chromosome-related databases, and the other is higher-level genomic markers, including genes and motifs.

For k-mer-related features, Kmer-db (v1.9.2) (34) was used to calculate the percentage of shared k-mers between input sequence and k-mer databases, generating the features (i) r_k18 (percentage of k-mers shared with the chromosome database), (ii) rmp_k18 (with the chromosome-minus-plasmid database), (iii) p_k25 (with plasmid database), and (iv) pmr_k25 (with the plasmid-minus-chromosome database). In addition, (v) *rp* (a ratio measures the bias of an input sequence as being toward chromosome or plasmids) calculated by the equation *rp* = *r_k18*/(*r_k18* + *p_k25*), A higher *rp* indicates that the input sequence is likely biased toward chromosomes.

The higher-level genomic markers include gene-based features and motif-based features. The gene-based markers used by Plasmer include replicon distribution score (RDS), conjugation, mobilization, replication, and AMR genes. The RDS is a metric introduced by Platon measuring replicon distribution of marker protein sequences (MPS) in the MPS database. It calculates an RDS value for each protein sequence, where a positive value means the protein tends to distribute on plasmids and a negative value means it distributes on chromosomes. For each input sequence, the average RDS of all proteins on the sequences was used. The conjugation, mobilization, and replication genes are known to be enriched on plasmids, while the AMR genes are frequently distributed on plasmids, which may also help determine the origin of contigs. To generate these features, protein sequences were first extracted using Prodigal (v2.6.3) (35). The hmmsearch program in HMMER (v3.3.2) (36) was used to search the motifs of conjugation, mobilization, AMR, and replication. The alignment score and expectation value (E-value) were extracted, and the maximum values of all extracted genes on each input sequence were used, generating the features (vi) ConjMaxScore (maximum alignment score of conjugation genes on input sequence), (vii) ConjMaxEvalue (maximum –log[E-value] of conjugation genes), (viii) MobiMaxScore (maximum alignment score of mobilization genes), (ix) MobiMaxEvalue (maximum –log[E-value] of mobilization genes), (x) AMRMaxScore (maximum alignment score of AMR genes), (xi) AMRMaxEvalue (maximum –log[E-value] of AMR genes), (xii) RepMaxScore (maximum alignment score of replication genes), and (xiii) RepMaxEvalue (maximum –log[E-value] of replication genes). Meanwhile, the presence or absence of conjugation, mobilization, AMR, and replication genes was determined whether maximum –log(E-value) was ≥50 (present and marked “1”) or not (absent and marked “0”), which consequently generated binary features: (xiv) hasConjugation (has conjugation genes), (xv) hasMobilization (has mobilization genes), (xvi) hasAMR (has AMR genes), and (xvii) hasReplication (has replication genes). For RDS-related features, DIAMOND (v2.0.15.153) (37) was used to search the MPS database (with RDS value) downloaded from Zenodo (doi: 10.5281/zenodo.3349651), and the average RDS scores for each input sequence were calculated as describe in the Platon manuscript (18), which generated the features (xviii) RDSAvgScore (average RDS score of genes on input sequence) and (xiv) RDSMaxScore (maximum RDS score of genes), as well as (xx) rdsBias (the RDS bias of each sequence), where rdsBias is 1 if the average RDS is ≥5, rdsBias is −1 if the average RDS is ≤–10, and rdsBias is 0 if the average RDS is >–10 and RDS <5.

The motif-based markers used by Plasmer include *oriT* and rRNA. *oriT* represents the origin of transfer, which is a unique marker for plasmids. The rRNA is exclusively located on chromosomes. BLASTN (v2.12.0) (38) was used to search the *oriT* motif in input sequences, which produced the features (xxi) OritMaxEvalue (maximum –log[E-value] of *oriT*) and (xxii) hasOriT (presence; and marked “1” if the maximum –log[E-value] is ≥50; otherwise marked “0”). cmscan (v1.1.4) (39) was used to search rRNAs in input sequences, which produced the features (xxiii) rRNAMaxScore (maximum alignment score of rRNA motifs), (xxiv) rRNAMaxEvalue (maximum –log[E-value] of rRNA motifs), and (xxv) hasrRNA (presence; marked “1” if the maximum –log[E-value] is ≥50; otherwise marked “0”). Meanwhile, considering that multiple marker genes are rarely simultaneously seen on input sequences, we also added a (xxvi) maxEvalue (maximum of all E-value-derived features described above except rRNAMaxEvalue), which measures the presence of any plasmid-related markers. Together, all 26 features described above were combined for model training and prediction. The reference databases used to extract conjugation, mobilization, replication, AMR, and rRNA genes were downloaded from Zenodo (doi: 10.5281/zenodo.3349651).

### Training set, feature selection, and model construction.

To construct the prediction model, a total of 31,897 complete bacterial genomes were downloaded from the NCBI GenBank database (Table S2). Plasmid sequences were selected with the keyword “plasmid” in the sequence name, and the rest were chromosomal sequences. Considering that the sequences of longer than 500 kb rarely originated from plasmids, as those often encode genetic features hindering the assembly of larger sequences (18), chromosomal sequences shorter than 500 kb were filtered out to avoid plasmid contamination as much as possible. To ensure that Plasmer learned from contigs of various lengths, we slid all chromosome and plasmid sequences into various window sizes, including 500 kb, 200 kb, 100 kb, 50 kb, 20 kb, 10 kb, 5 kb, 2 kb, 1 kb, and 500 bp. The overlapping lengths for adjacent windows were 50 kb, 20 kb, 10 kb, 5 kb, 2 kb, 1 kb, 500 bp, 200 bp, 100 bp, and 50 bp, respectively (Table S3). To generate a training set with approximately 1 million sequences of mixed lengths, we sampled 80% of the sequences (up to 100,000 sequences were used if the number of sequences exceeded 100,000) from each window size. Finally, a total of 1,573,876 sequences, including 900,000 chromosome fragments and 673,876 plasmid fragments, were labeled and used as the training set for model training.

Based on the 1,573,876 sequences, the importance and feature selection were performed with the R package Boruta (40) (Fig. S1C). The random forest classifier was constructed using the R package randomForest (41) (*n*trees = 500) by randomly sampling 2/3 sequences as the training set and the remaining 1/3 as the test set, and the receiver operating characteristic (ROC) curves were illustrated using the R packages ROCR (42) and pROC (43). The model construction was conducted 100 times to check the consistency and stability. For a query sequence, the detailed workflow is illustrated in Fig. S1B.

### Taxonomy classification of predicted plasmids.

A customized Kraken2 database was constructed using all plasmids in the NCBI RefSeq database for taxonomy classification using kraken2-build. Input sequences classified as plasmid were further classified using Kraken 2 (v2.1.2) (44) using the customized Kraken 2 database for taxonomy.

### Performance benchmarks.

**(i) Prediction using available tools.** Platon (v1.6) (18) was installed using anaconda, and the Platon database was downloaded from https://doi.org/10.5281/zenodo.3349651. Default parameters were used for all Platon predictions.

The mlplasmids program (21) was installed using anaconda. The cutoff of posterior probability was set to 0.7. All three models, Enterococcus faecium, Klebsiella pneumoniae, and Escherichia coli, were used. Because mlplasmids has no generic model for all species, it was omitted in the performance comparison of sliding sequences and real sequencing data of Deeplasmid and PlasFlow. In the simulated contigs and real sequencing data of the Platon and mlplasmids manuscripts, a sequence was considered a plasmid sequence if it was predicted positive in the specific model.

PlasFlow (20) was installed using anaconda. As PlasFlow contains an “unclassified” class, which is not a true binary classifier, the input sequences predicted as unclassified were dropped, and only chromosome and plasmid sequences were retained. The calculated metrics without “unclassified” are shown in the figures and supplemental tables. Conversely, we also demonstrate metrics in the supplemental tables in the case that “unclassified” was treated as chromosome sequences. Default parameters were used for all PlasFlow predictions.

RFPlasmid (23) was installed using anaconda. The “generic” species category was used for the sake of comparability with other tools and compatibility with the randomly sampled sequences regardless the species. For benchmarks on simulated and real data, species-specific models were used if available and marked “RFPlasmid-specific” in plots.

Deeplasmid (24) was obtained using the Docker Hub, and the graphics processing unit (GPU)-based version was used. Default parameters were used for all Deeplasmid predictions. As the run time for Deeplasmid was much longer than that of the other tools as the number of sequences grew, to finish tests in a reasonable runtime, we downsampled 1% for 500 kb, 0.7% for 200 kb, 0.1% for other chromosome lengths, and 3% for each plasmid length. For simulated and real data benchmarks, the complete data were used without sampling.

The benchmarks for Platon, mlplasmids, PlasFlow, and RFPlasmid were finished on a small cluster with 4 nodes, each equipped with dual-socket AMD EPYC 7702 64-core processor and 512 GB of memory. The benchmarks for Deeplasmid were finished on a workstation equipped with an Intel Core i7-9700K CPU, 32 GB of memory, and NVIDIA RTX2080 SUPER 8GB. All the benchmarking flowcharts are illustrated in Fig. S1D.

**(ii) Performance on different input lengths.** To evaluate the performance and applicability of Plasmer on different input lengths, we used the sliding sequences of all complete bacterial genomes except those used in training set as input for benchmarking. The tests were also done in different window sizes from 500 kb down to 500 bp. By defining the plasmids as positive and chromosomes as negative, a confusion matrix was built counting the number of true positives (*tp*), true negatives (*tn*), false positives (*fp*), and false negatives (*fn*), upon which the metrics sensitivity (or plasmid accuracy) (*tp*/[*tp* + *fn*]), specificity (or chromosome accuracy) (*tn*/[*tn* + *fp*]), accuracy ([*tp* + *tn*]/[*tp* + *tn* + *fp* + *fn*]), precision (*tp*/[*tp* + *fp*]), FNR (*fn*/[*tp* + *fn*]), FPR (*fp*/[*tn* + *fp*]), FOR (*fn*/[*fn* + *tn*]), FDR (*fp*/[*fp* + *tp*]), NPV (*tn*/[*fn* + *tn*]), and F1-score (2 × [sensitivity × precision]/[sensitivity + precision]) were calculated. These metrics were calculated and plotted for each length and each method for parallel comparison. Since sliding windows are small fragments of assembled contigs with identical lengths, it creates a huge number of sequences if the windows are small. As a result, the number of sliding sequences from chromosomes was much higher than the number of plasmids and was severely unbalanced, which greatly reduced the reliability of metrics that relied on balanced sample size, such as precision, accuracy, FDR, and F1-score. Therefore, to demonstrate the unbiased metrics for all tested methods, we simultaneously conducted additional benchmarks on balanced numbers of sliding windows from chromosomes and plasmids by downsampling sequences from chromosomes. The downsampled data were used only in the benchmark of different input length, not in model construction.

**(iii) Performance on simulated assemblies of isolate genomes.** To evaluate the performance and applicability of Plasmer on short-read assemblies, we used wgsim (v1.14) (https://github.com/lh3/wgsim) to simulate the sequencing reads from 552 complete Klebsiella pneumoniae genomes, 1,718 complete Escherichia coli genomes, and 123 complete Enterococcus faecium genomes (Table S4). The simulation parameter was set to “-e 0 -d 500 -N 1000000 -1 150 -2 150”. For each complete genome, the simulated reads were assembled using MEGAHIT (v1.2.9) (45) with default parameters. The contigs that shared <95% of k-mers with the original reference genome were considered mis-assembled contigs and were discarded. The cleaned contigs were then labeled “chromosome” or “plasmid” according to their bias of the percentage of shared k-mers with the chromosomes and plasmids in the original genome using Kmer-db (v1.9.2) (34). SeqKit (v2.2.0) (46) was used to calculate the length of labeled contigs. The labeled contigs were used as input for all tested methods. Benchmarks were evaluated as described above.

**(iv) Performance on *de novo* assemblies from real sequencing data.** To evaluate the performance and applicability of Plasmer on *de novo* assemblies from real sequencing data, we downloaded and assembled the Illumina short reads used in mlplasmids, PlasFlow, Platon, and Deeplasmid (Tables S5 to S7). For libraries with a corresponding reference genome, we *de novo*-assembled the short reads using MEGAHIT (v1.2.9) (45) and labeled the contigs as chromosome or plasmid as described above for simulated assemblies. For libraries without a reference genome but simultaneously sequenced by Nanopore, we *de novo*-assembled the Nanopore long reads using Flye (v2.9) (47) and defined the chromosome and plasmid contigs according to the assembly_info.txt file in the results and the sequence similarity according to PLSDB. For example, 3 contigs were assembled; one was several Mb long, and the other two were tens of kb. If the shorter two were similar to PLSDB (BLASTN; coverage, ≥50%), they were assigned to plasmids, while the long one was assigned to chromosomes. Next, contigs that were *de novo* assembled using MEGAHIT (v1.2.9) (45) were labeled according to the label of the long-read assemblies. Since no Illumina data were available for the Yersinia ruckeri isolate, we simulated short reads from the Nanopore assembly (Tables S12 and S13). Benchmarks were evaluated as described above for simulated assemblies.

To further evaluate the performance of Plasmer on new genomes and multiple species, we downloaded 535 complete genomes of multiple species recently published in NCBI (between 1 July 2022, and 15 April 2023) (Table S14). These genomes were released after current training of Plasmer and thus were not included in the database and training set. These genomes were sequenced with both short (Illumina) and long (PacBio/Nanopore) reads, which allowed use of the high-quality reference genome to label the chromosome and plasmid in the short-read assemblies. The short-read contigs were *de novo* assembled using MEGAHIT (v1.2.9) (45) for each genome, and contigs that shared <95% of k-mers with the downloaded reference genome were considered mis-assembled contigs and were discarded. The cleaned contigs were then labeled “chromosome” or “plasmid” according to the bias of their percentage of shared k-mers with chromosomes or plasmids in the reference genome as determined using Kmer-db (v1.9.2) (34). SeqKit (v2.2.0) (46) was used to calculate the length of labeled contigs. The labeled contigs were predicted using Plasmer and other tools. Benchmarks were evaluated as described above.

### Data availability.

Plasmer is open-source software under active development and is freely available at https://github.com/nekokoe/Plasmer. A docker container version is available on Docker Hub at https://hub.docker.com/repository/docker/nekokoe/plasmer. A conda package is also available at https://anaconda.org/iskoldt/plasmer; users can install Plasmer with the command “conda install -c iskoldt -c bioconda -c conda-forge -c defaults plasmer.” The databases can be downloaded by following to the instructions on the GitHub repository or from Zenodo at https://doi.org/10.5281/zenodo.7030674.
