# Supplementary material for: Plasmer: an Accurate and Sensitive Bacterial Plasmid Prediction Tool Based on Machine Learning of Shared k-mers and Genomic Features
Source: Microbiol Spectr. 2023 May 16;11(3):e04645-22. doi: 10.1128/spectrum.04645-22 (PMC10269668; doi:10.1128/spectrum.04645-22)
Supplement: Supplemental file 2 — Fig. S1 to S4 and legends of Tables S1 to S15. Download spectrum.04645-22-s0002.pdf, PDF file, 4.6 MB [file spectrum.04645-22-s0002.pdf]

Supplementary Figure S1. The detail workflow of our work. (A) The construction of k-mer database. (B) The predictable flow chart of Plasmer. (C) The detail of model construction. (D) The detail of performance evaluation.

Supplementary Figure S2. The distribution of shared k-mers between nc, np and r, p, rmp, pmr databases at k from 17 to 27, representatively. The x axis represents different k; the y axis represents percentage of shared k-mers. The nc and np represent the chromosomes and plasmids of 31,897 complete bacterial genomes in NCBI, respectively. The r, p, pmr, rmp represent the k-mer database from chromosomes of representative genomes, the k-mer database from plasmids of PLSDB, plasmid-unique k-mer database (Plasmids database Minus k-mers from chromosomes of Representative genomes) and the chromosome-unique k-mer database (Representative chromosome database Minus k-mers from PLSDB), respectively. (A) The distribution of shared k-mers between nc, np and r, p databases. (B) The distribution of shared k-mers between nc, np and rmp, pmr databases.

Supplementary Figure S3. The PCoA plot of the 1,573,876 sliding sequences used to train the model based on r_k18, p_k25, rmp_k18 and pmr_k25 features. Orange color represents plasmid and lake-green represents chromosome.

Supplementary Figure S4. The distribution of all features in chromosome and plasmid of 1,573,876 sliding sequences.

Supplementary Table S1. The information of 2,935 representative complete genomes in NCBI.

Supplementary Table S2. The information of 31,897 complete genomes in NCBI.

Supplementary Table S3. Statistic of sliding windows with different window size.

Supplementary Table S4. The genomes used for simulated assemblies of *K. pneumoniae*, *E. coli* and *E. faecium*.

Supplementary Table S5. Real sequencing data for benchmark used in PlasFlow

manuscript.

Supplementary Table S6. Real sequencing data for benchmark used in mlplasmids manuscript.

Supplementary Table S7. Real sequencing data for benchmark used in Platon and Deeplasmid manuscripts.

Supplementary Table S8. Performance benchmark on remaining sliding sequences of 31,897 complete bacterial genomes. Deeplasmid can only predict sequences longer than 1 kb. Platon excluded all the sequences shorter than 1 kb.

Supplementary Table S9. Performance benchmark on remaining sliding sequences of 31,897 complete bacterial genomes in balanced sample size of chromosome and plasmid. Deeplasmid can only predict sequences longer than 1 kb. Platon excluded all the sequences shorter than 1 kb.

Supplementary Table S10. Performance benchmark on simulated assemblies. mlplasmids and Deeplasmid can only predict sequences longer than 1 kb.

Supplementary Table S11. Performance benchmark on real sequencing data. mlplasmids and Deeplasmid can only predict sequences longer than 1 kb.

Supplementary Table S12. Statistics of *de novo* assembly genomes based on ONT sequencing data from Platon and Deeplasmid manuscripts.

Supplementary Table S13. The assembly information of *de novo* assmebled genomes based on ONT sequencing data from Platon and Deeplasmid manuscripts.

Supplementary Table S14. The summary of 535 recently published genomes of multiple species.

Supplementary Table S15. Performance benchmark on de novo assembled sequences from 535 recently published genomes of multiple species.

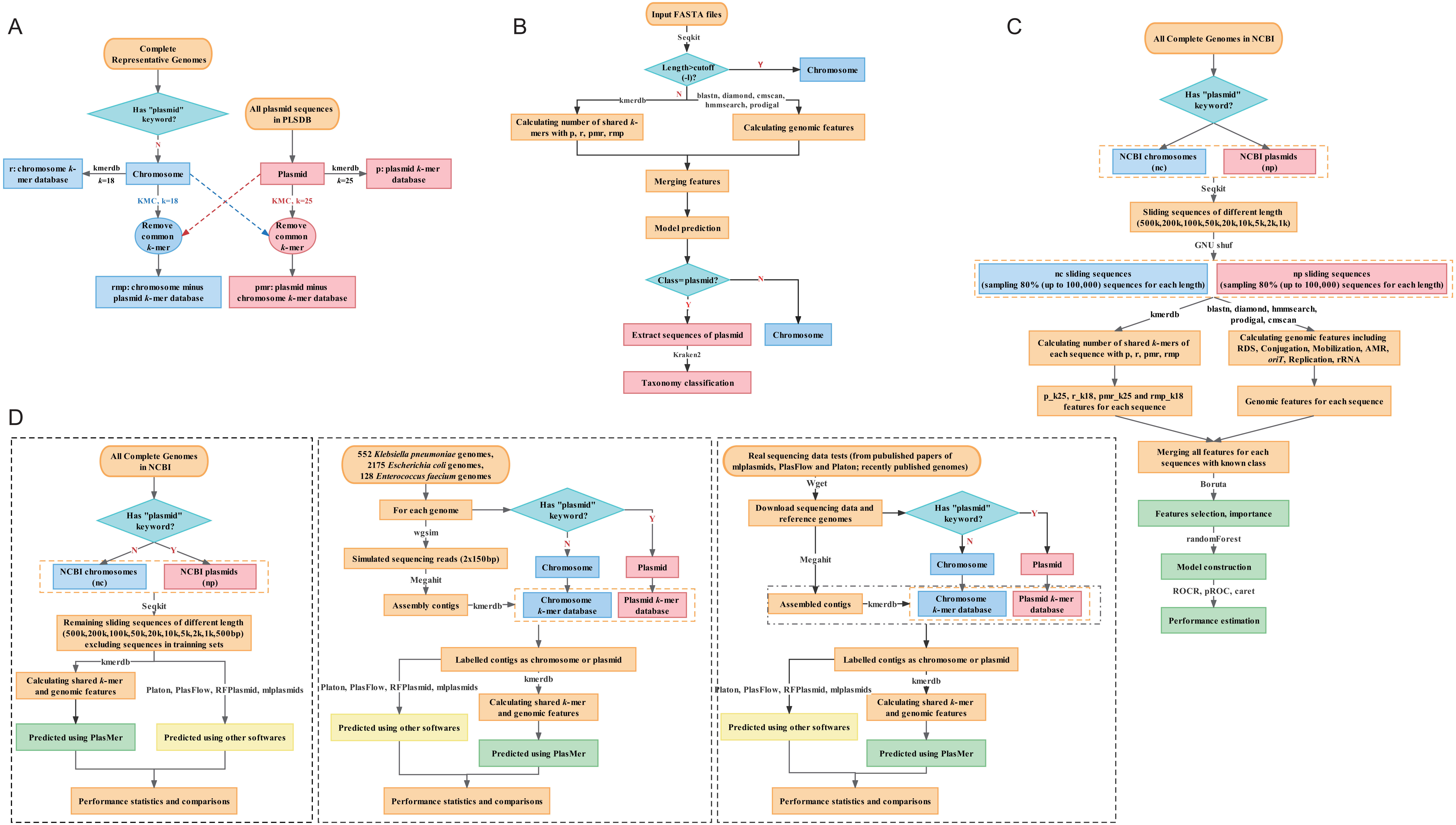

Supplementary Figure S1. The detail workflow of our work. (A) The construction of k-mer database. (B) The predictable flow chart of Plasmer. (C) The detail of model construction. (D) The detail of performance evaluation.

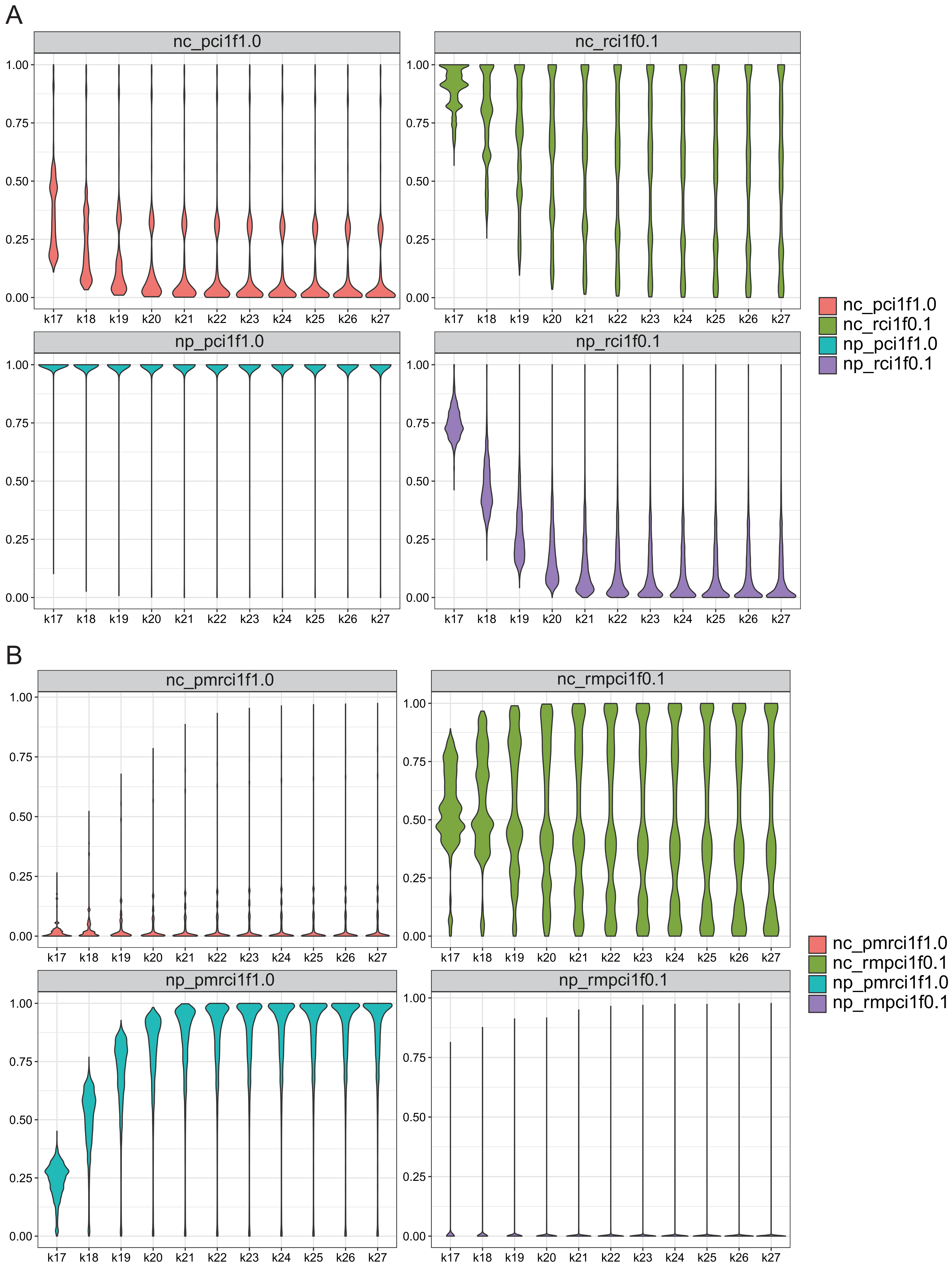

Supplementary Figure S2. The distribution of shared k-mers between nc, np and r, p, rmp, pmr databases at k from 17 to 27, representatively. The x axis represents different k; the y axis represents percentage of shared k-mers. The nc and np represent the chromosomes and plasmids of 31,897 complete bacterial genomes in NCBI, respectively. The r, p, pmr, rmp represent the k-mer database from chromosomes of representative genomes, the k-mer database from plasmids of PLSDB, plasmid-unique k-mer database (Plasmids database Minus k-mers from chromosomes of Representative genomes) and the chromosome-unique k-mer database (Representative chromosome database Minus k-mers from PLSDB), respectively.(A) The distribution of shared k-mers between nc, np and r, p databases. (B) The distribution of shared k-mers between nc, np and rmp, pmr databases.

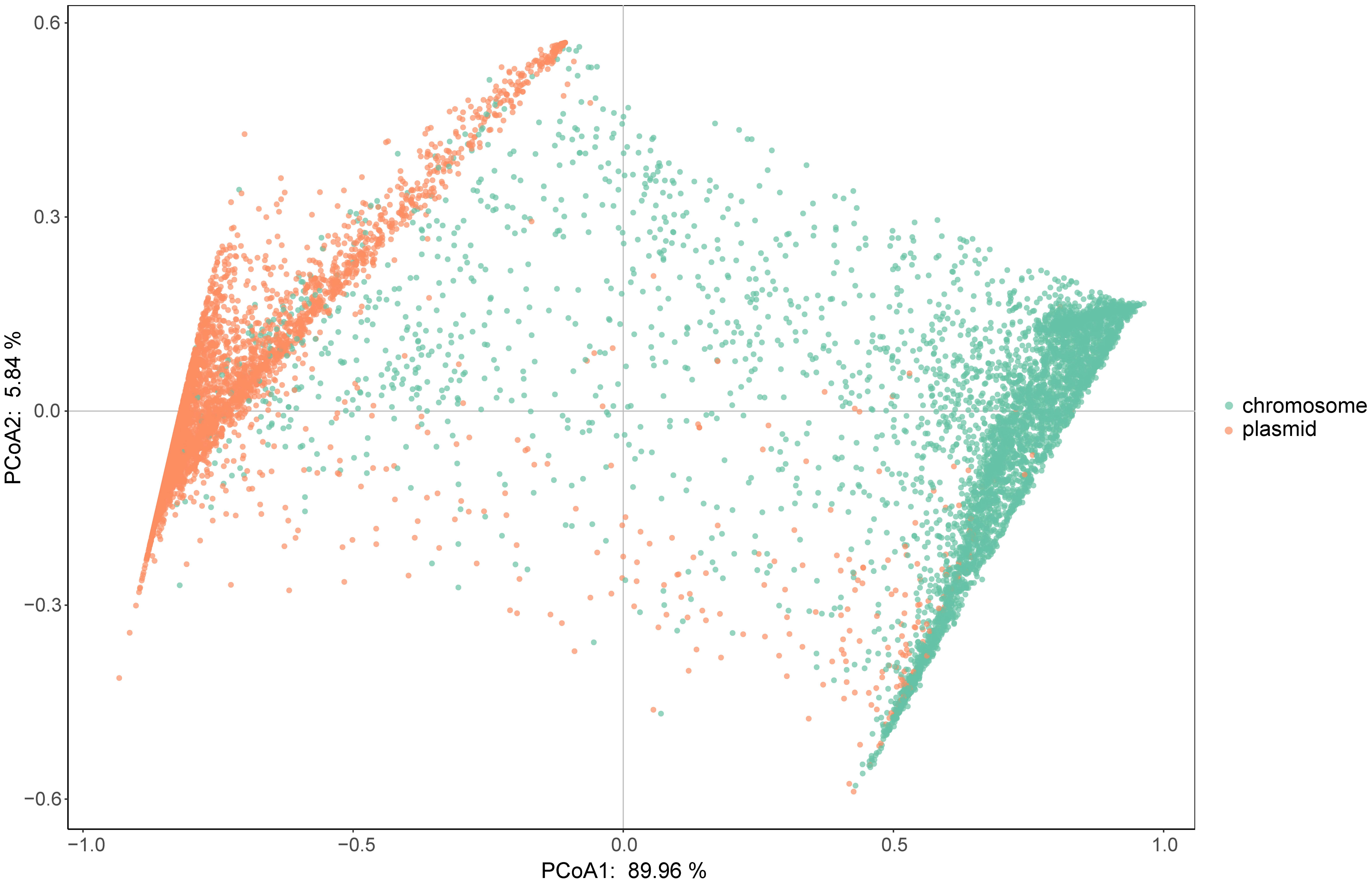

Supplementary Figure S3. The PCoA plot of the 1,573,876 sliding sequences used to train the model based on r_k18, p_k25, rmp_k18 and pmr_k25 features. Orange color represents plasmid and lake-green represents chromosome.

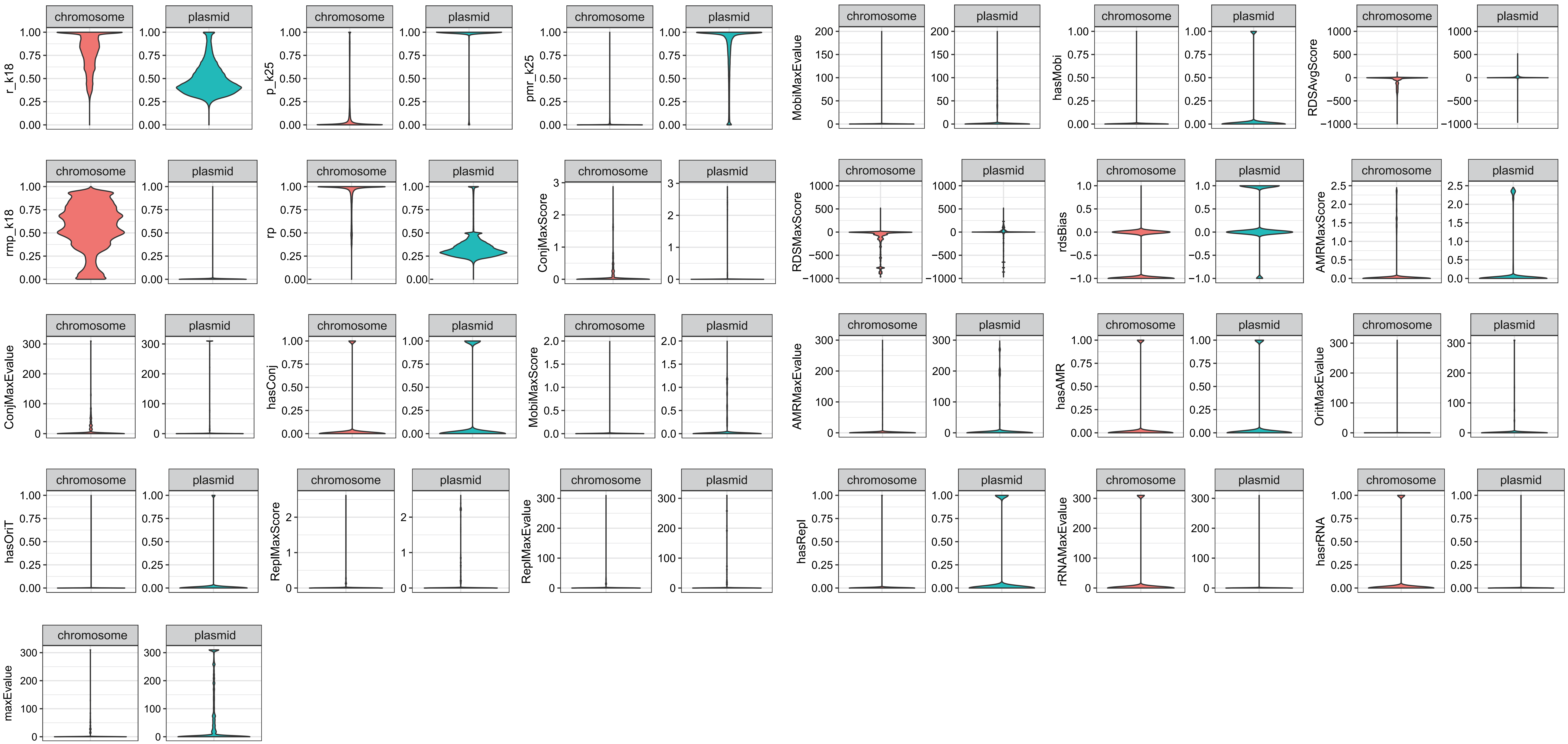

Supplementary Figure S4. The distribution of all features in chromosome and plasmid of 1,573,876 sliding sequences.
